# Magnesium- a Forgotten Element: Phenotypic Variation and Genome Wide Association Study in Turkish Common Bean Germplasm

**DOI:** 10.3389/fgene.2022.848663

**Published:** 2022-05-02

**Authors:** Faheem Shehzad Baloch, Muhammad Azhar Nadeem, Ferit Sönmez, Ephrem Habyarimana, Zemran Mustafa, Tolga Karaköy, Gönül Cömertpay, Ahmad Alsaleh, Vahdettin Çiftçi, Sangmi Sun, Gyuhwa Chung, Yong Suk Chung

**Affiliations:** ^1^ Faculty of Agricultural Sciences and Technologies, Sivas University of Science and Technology, Sivas, Turkey; ^2^ Department of Seed Science and Technology, Faculty of Agriculture, Bolu Abant Izzet Baysal University, Bolu, Turkey; ^3^ International Crops Research Institute for the Semi-Arid Tropics, Patancheru, India; ^4^ Eastern Mediterranean Agricultural Research Institute, Adana, Turkey; ^5^ Molecular Genetic Laboratory, Science and Technology Application and Research Center, Institute for Hemp Research, Yozgat Bozok University, Yozgat, Turkey; ^6^ Department of Field Crops, Faculty of Agriculture, Bolu Abant Izzet Baysal University, Bolu, Turkey; ^7^ Department of Biotechnology, Chonnam National University, Chonnam, South Korea; ^8^ Department of Plant Resources and Environment, Jeju National University, Jeju, South Korea

**Keywords:** phaseolus vulgaris, food legume, mg contents, DArTseq, GWAS

## Abstract

Magnesium (Mg) is the fourth most abundant element in the human body and plays the role of cofactor for more than 300 enzymatic reactions. In plants, Mg is involved in various key physiological and biochemical processes like growth, development, photophosphorylation, chlorophyll formation, protein synthesis, and resistance to biotic and abiotic stresses. Keeping in view the importance of this element, the present investigation aimed to explore the Mg contents diversity in the seeds of Turkish common bean germplasm and to identify the genomic regions associated with this element. A total of 183 common bean accessions collected from 19 provinces of Turkey were used as plant material. Field experiments were conducted according to an augmented block design during 2018 in two provinces of Turkey, and six commercial cultivars were used as a control group. Analysis of variance depicted that Mg concentration among common bean accessions was statistically significant (*p* < 0.05) within each environment, however genotype × environment interaction was non-significant. A moderate level (0.60) of heritability was found in this study. Overall mean Mg contents for both environments varied from 0.33 for Nigde-Dermasyon to 1.52 mg kg^−1^ for Nigde-Derinkuyu landraces, while gross mean Mg contents were 0.92 mg kg^−1^. At the province level, landraces from Bolu were rich while the landraces from Bitlis were poor in seed Mg contents respectively. The cluster constellation plot divided the studied germplasm into two populations on the basis of their Mg contents. Marker-trait association was performed using a mixed linear model (Q + K) with a total of 7,900 DArTseq markers. A total of six markers present on various chromosomes (two at Pv01, and one marker at each chromosome i.e., Pv03, Pv07, Pv08, Pv11) showed statistically significant association for seed Mg contents. Among these identified markers, the DArT-3367607 marker present on chromosome Pv03 contributed to maximum phenotypic variation (7.5%). Additionally, this marker was found within a narrow region of previously reported markers. We are confident that the results of this study will contribute significantly to start common bean breeding activities using marker assisted selection regarding improved Mg contents.

## 1 Introduction

Incensement of mineral contents in staple food to provide the recommended daily intake is crucial to fight nutrient deficiencies in the diets of humans as more than half of the world population receives insufficient essential mineral elements (Frossard et al., 2000; White and Broadley, 2009). Magnesium is the fourth most abundant mineral in human body and serves as a cofactor for more than 300 enzymes. It has a crucial role in protein synthesis, muscle contraction nerve transmission, blood pressure regulation, glucose metabolism, and signal transduction (Gröber et al., 2015). Magnesium deficiency is linked with insulin resistance, diabetes, cardiovascular diseases, stroke, and obesity (Cakmak, 2013; Bertinato et al., 2015; Gröber et al., 2015). The adult human body contains around 25 g Mg (Elin, 1987) and the estimated average daily requirement (EAR) of Mg is 265 and 350 mg for adult females and males, respectively (Rude, 1998). In plants, Mg plays many important roles in metabolism and its deficiency causes reduction in growth and yield. Since up to 35% of Mg is found in chloroplasts, chlorosis and yellowing in leaves is a typical symptom of its deficiency (Farhat et al., 2016). It is also utilized as an adaptation against aluminium toxicity where it is released from the roots to chelate aluminium ions in the soil (Cakmak and Yazici, 2010).

The Fabaceae or Leguminosae is one of the most important family in the kingdom Plantae and individuals (legumes) of this family are a great source of high quality protein, minerals, and vitamins. Legumes are multi-benefit crops as they contribute significantly to atmospheric nitrogen fixation, increase the high quality organic matter content in soil, and improve water retention. These benefits have increased the importance of legumes for sustainable agriculture under a changing climate (Stagnari et al., 2017). Among various legumes, common bean is considered as a “grain of hope” due to its nutritional potential (Nadeem et al., 2021a). Currently, this crop is cultivated all over the world and a total of 34 mha area was under common bean cultivation globally during 2020, resulting in a production of 27.5 mtones (FAO, 2022). Earlier studies confirmed that common bean was originated and domesticated in Mesoamerica and arrived in Europe through Columbian exchange (Rodiño et al., 2006; Gioia et al., 2013; Nadeem et al., 2021a), and then to Turkey through Ottoman traders. Turkey is considered a hotspot for agricultural biodiversity for most of the crops we use in our kitchen today (Nadeem et al., 2018; Nadeem et al., 2020a). Since common bean is not native to Turkey, however, it has a unique place in Turkish agriculture and hundreds of common bean landraces have emerged over time in different parts of Turkey due to variations in the agricultural practices, topography, and taste preference of local people (Nadeem et al., 2018; Nadeem et al., 2020a).

In Turkey, common bean is one of the important sources of protein, minerals, and calories after cereals as Turkish people use common bean at least once in a week either as unripe pods as vegetables, dry seeds, or in the form of salads (Nadeem et al., 2020a). Annual common bean production in Turkey in dry or fresh form was around 279,518 tons, making Turkey 3rd largest producer of the common bean in the world (Yeken et al., 2019) and 1st largest producer in the Mediterranean region (Yeken et al., 2019). An increase in common bean production has been recorded in Turkey in the last decade. This increase in common bean production can possibly be due to a good number of breeding activities carried out in Turkey. To date, a good number of common bean cultivars (200 fresh and 39 dry) have been registered in Turkey (Variety registration and seed certification center; www.tarimorman.gov.tr). As common bean has a critical place in Turkish diets and around the world, so breeding common bean cultivars with high Mg contents is crucial for fighting mineral malnutrition.

Breeding methodologies developed rapidly in the last few decades due to increasing demand of the safe foods for an increasing population of the world. The first step for breeding the common bean cultivars for high Mg contents is to evaluate the natural and ancestral germplasm, particularly from its area of diversity (Baloch et al., 2014). Various agencies of the world engaged with biodiversity have put emphasis on the collection and characterization of the germplasm, as they harbour novel alleles for the traits of interest (Baloch and Nadeem, 2022; Nadeem et al., 2020a,b). Characterization of common bean germplasm is crucial to explore the variations in order to select the elite genotypes having high Mg contents and to identify the genetic regions controlling the Mg contents in common bean seed. There is plentiful variability of seed Mg concentration in common bean. Variation of Mg contents in the seeds of common bean germplasm from various parts of the world is well documented in the earlier studies (Barampama and Simard, 1993; Sangronis and Machado, 2007; Wang et al., 2010; Akond et al., 2011; Brigide et al., 2014; Yeken et al., 2019). In our previous study, we characterized 80 Turkish common bean accessions to explore mineral elements diversity and identified Mg contents in a range of 0.63–0.94 (mg kg^−1^). Magnesium contents harbored by the common bean germplasm could be utilized in common bean breeding to increase Mg concentrations in edible parts as common bean is frequently used in the human diet in all parts of the world (Yeken et al., 2019).

During germplasm characterization for yield and mineral traits, environment and genotype interaction should be considered one of the most important factors, as the same plant can be affected hugely from its surroundings (Shrestha et al., 2012; Misra et al., 2020). To effectively breed crops with advanced phenotypic performances, knowledge about its adaptations and its reaction in different growing conditions and environments should be elucidated (Falconer et al., 1996). Both environmental and genetic factors affect the accumulation of Mg concentration in dry and fresh common bean seeds (Moraghan et al., 2006). Therefore, breeding common bean cultivars require the characterization of the germplasm under various environmental conditions.

Genome-wide association studies (GWAS) are a powerful genomic tool for the identification of linked markers using variation harbored by natural germplasm. GWAS, a strong structural genomics technique to screen large number of accessions using next generation sequencing (NGS) based markers covering the whole genome of common bean, has been used to identify the linked marker for various traits of agricultural and nutritional importance with high resolution (Nadeem et al., 2020b; Nadeem et al., 2021b). However very few studies evaluated the germplasm for Mg contents variations in common bean and identified QTLs/linked-markers. Delfini et al. (2021) used a Brazilian germplasm, Gunjaca et al. (2021) used a Croatian germplasm, Blair et al. (2016) and Casañas et al. (2013) used RIL populations for the identification of QTLs/linked-markers associated with Mg contents. Despite the importance of the Mg for human health and crop production, little research work is documented for breeding the common bean for Mg concentration. Therefore, Mg is referred to as “A Forgotten Element”. However, under a changing climate and a rapidly increasing world population scenario, Mg deficiency is becoming a critical limiting factor for common bean production and indirectly for human health.

In the present research, mini core collection of 183 common bean genetic resources collected from 19 provinces of Turkey was established to identify the chromosomal regions associated with seed Mg contents. This study also aimed to check whether the markers identified in our study fall within the same genetic region or whether new QTLs for seed Mg contents are available in the Turkish common bean germplasm.

## 2 Materials and Methods

### 2.1 Plant Material

During this study, a total of 177 common bean landraces collected from 19 provinces of Turkey and six commercial cultivars were used as plant material. The studied germplasm was collected from a farmer’s field and the core collection was established at Bolu Abant Izzet Baysal University. Detailed information about this plant material can be found in Supplementary Table S1. This material was sown at the research and implementation area of Bolu Abant Izzet Baysal University and single plant selection was performed for two consecutive years during 2014 and 2015. In later years, seed multiplication was performed to get a high enough quantity of seeds for each accession for further genetics and breeding studies.

### 2.2 Field Experimentation

Field experiments were conducted at two geographical locations; Bolu and Sivas during 2018 according to Augmented Block design. A total of six commercial cultivars (Akman, Goynuk, Karacaşehir, Onceler, Goksun, and Akdag) were used as control groups. These cultivars were repeated in each block to standardize the mean of all accession. Sowing was performed on 12th and 17th april 2018 in Sivas and Bolu locations, respectively. All accessions were sown in a single row of 3 m length with 50 cm row to row and 10 cm plant to plant distance. All standard agronomic practices were followed during this study. Detailed information about the field experiments and applied agricultural practices can be found in our previous work (Nadeem et al., 2020a).

### 2.3 Phenotypic Analysis for Mg Contents of Common Bean Germplasm

Harvesting was performed at 90% pod maturity and seed samples were taken from each accession in three replicates. Seed Mg contents were investigated according to the methodology described by Yeken et al. (2019). Firstly, seeds were grinded and a fine powder was obtained. A total of 0.2 g seed sample from each accession was digested with 5 ml of concentrated nitric acid (65%) and 2 ml of hydrogen peroxide (35%) in a closed microwave digestion system (ETHOS EASY, Milestone, Italy) (Bremner, 1965; Seco-Gesto et al., 2007). After the completion of the digestion process, solutions were transferred to flasks and a final volume of 20.0 ml was maintained with ultra-pure water. This prepared solution was used for the investigation of seed Mg contents with Atomic Absorption Spectrophotometer (Shimadzu AA-7000). Seed Mg contents were repeated three times for each sample and recorded as mg kg^−1^.

### 2.4 Genotyping of Common Bean Germplasm

DNA was extracted from the single selected plants according to CTAB protocol of Doyle and Doyle (1990) with a specific protocol suggested by Diversity Arrays Technology (available at “http://www.diversityarrays.com/orderinstructions/plant-dnaextraction-protocol-for-dart/)” \o “http://www.diversityarrays.com/orderinstructions/plant-dnaextraction-protocol-for-dart/)” \h www.diversityarrays.com/orderinstructions/plant-dnaextraction-protocol-for-dart/). Quality and quantity of DNA was calculated on the agarose gel (0.8%). DNA was diluted to a final concentration of 50 ng/ul and DNA samples were sent to diversity array technology (http://www.diversityarrays.com/) for DArTseq analysis based genotyping by sequencing technology (GBS). The detailed information about GBS analysis for DArTseq markers could be traced from our previously published study (Nadeem et al., 2018).

### 2.5 Statistical Analysis

#### 2.5.1 Phenotypic Analysis

In this study, the sowing of the germplasm was performed in eight blocks, while six commercial cultivars were repeated eight times as a control. Repetition of commercial cultivars was used in the standardization of data and for the calculation of adjusted means. Analysis of variance (ANOVA) was conducted to get an idea about the effect of genotype and genotype × environment interaction for seed Mg contents in studied germplasm. Analysis of variance (ANOVA) was calculated utilizing these evaluated adjusted means. Firstly, ANOVA was calculated within environments and later ANOVA was performed across the environments using agricolae: an R package (De Mendiburu and Simon 2015). Mean, maximum, and minimum Mg contents for the studied environments were investigated through XLSTAT statistical software (www.xlstat.com). Frequency distribution and provinces based Mg contents were calculated through XLSTAT statistical software. The most stable common bean accessions for Mg contents were investigated through the online software “STABILITYSOFT” (Pour-Aboughadareh et al., 2019). The constellation plot for common bean accessions was constructed through JMP 14.1.0 statistical software (2018, SAS Institute Inc., Cary, NC, United States).

#### 2.5.2. Marker-Trait Investigation for Seeds Mg Contents

The Q-matrix and Kinship are basic requirements while performing bioinformatics analysis for GWAS studies as both are used to correct the population and family structure during the association analysis. Population structure of studied germplasm was performed previously and has been reported in our published study (Nadeem et al., 2018). Therefore, the required Q-matrix for GWAS analysis was evaluated from a previous study (Nadeem et al., 2018). Marker trait association was performed using mixed linear model approach (MLM, Q + K). Tassel 5.2.50 (https://tassel.bitbucket.io) program was used to investigate the kinship matrix according to the methodology of Bradbury et al. (2007). False discovery rate (FDR) and Bonferroni (*p* = 0.01) thresholds were used in the present study to investigate the significantly associated markers. A Manhattan plot was developed to visualize the statistically significant markers through R 3.4.1 statistical software (http://www.r-project.org/) by using qq-man R Package (Turner, 2014). A physical map was constructed for the identified linked DArTseq markers through R 3.4.1 statistical software to confirm whether they were present or not at same chromosomal region.

## 3 Results

### 3.1 Phenotypic Diversity

The results showed that there was a plentiful and continuous diversity for seed Mg concentrations among common bean accessions (Supplementary Table S2). The ANOVA results depicted that Mg concentration among common bean accessions was statistically significant within each environment (data now shown), however genotype × environment interaction was non-significant (Table 1). Heritability analysis showed a moderate level (0.60) of heritability (Table 1).

**TABLE 1:** Summary of analysis of variance in Turkish common bean germplasm.

Variables	Df	Sum Sq	Mean Sq	F value	Pr (>F)
Genotype	182	1,089.683	5.827182	1.958887	2.13E-08
GxE	183	238.4785	1.268,503	0.426424	1
Residuals	366	1,118.503	2.974741	NA	NA
Heritability			0.597895		

*NA: Not available.

**FIGURE 1:**
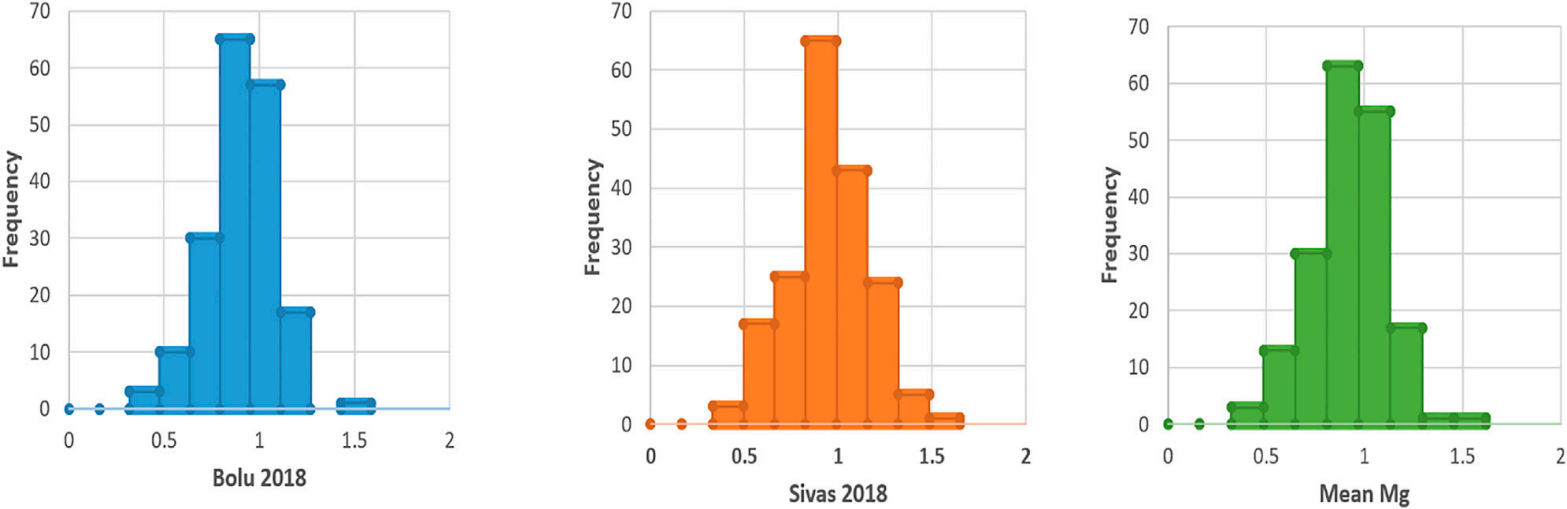
Frequency distribution of Mg contents in Turkish common bean seeds.

During 2018 at Bolu, Mg contents ranged from 0.32–1.49 mg kg^−1^ for Nigde-Dermasyon and Nigde- Derinkuyu landraces respectively, while mean Mg contents were 0.90 mg kg^−1^ (Table 2). Similarly, at Sivas, seed Mg contents ranged from 0.34–1.55 mg kg^−1^ for Nigde-Dermasyon and Nigde-Derinkuyu landraces respectively, while mean Mg contents were 0.95 mg kg^−1^. By taking the mean of both locations, overall Mg contents among accessions varied from 0.33–1.52 mg kg^−1^ for the above reported landraces, respectively. Frequency distribution of the Mg contents among common bean accessions is shown in Figure 1, which clearly depicted that most of the landraces with high Mg concentration in the common bean seeds had a value above 0.90 mg kg^−1^.

**TABLE 2:** Minimum, maximum and mean Mg (mg kg^−1^) contents in Turkish common bean germplasm under multi-year/environments.

Environment	Minimum	Maximum	Mean	Std. deviation
Bolu2018	0.320 (Nigde-Dermasyon)	1.490 (Nigde-Derinkuyu)	0.90	0.172
Sivas2018	0.340 (Nigde-Dermasyon)	1.550 (Nigde-Derinkuyu)	0.95	0.209
Mean	0.330 (Nigde-Dermasyon)	1.520 (Nigde-Derinkuyu)	0.92	0.186

**FIGURE 2:**
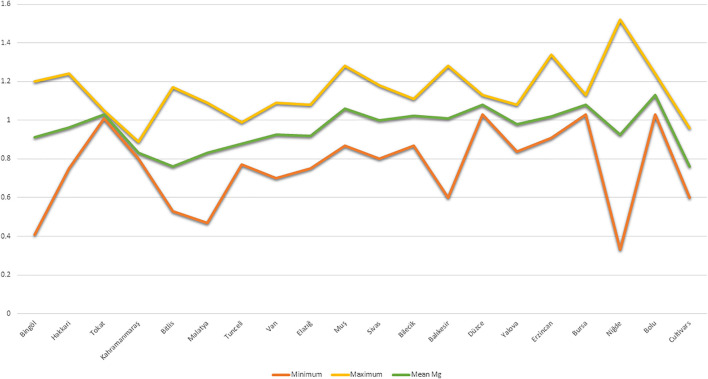
Variation of seed Mg contents in Turkish common bean germplasm on the basis of their collection provinces.

**FIGURE 3:**
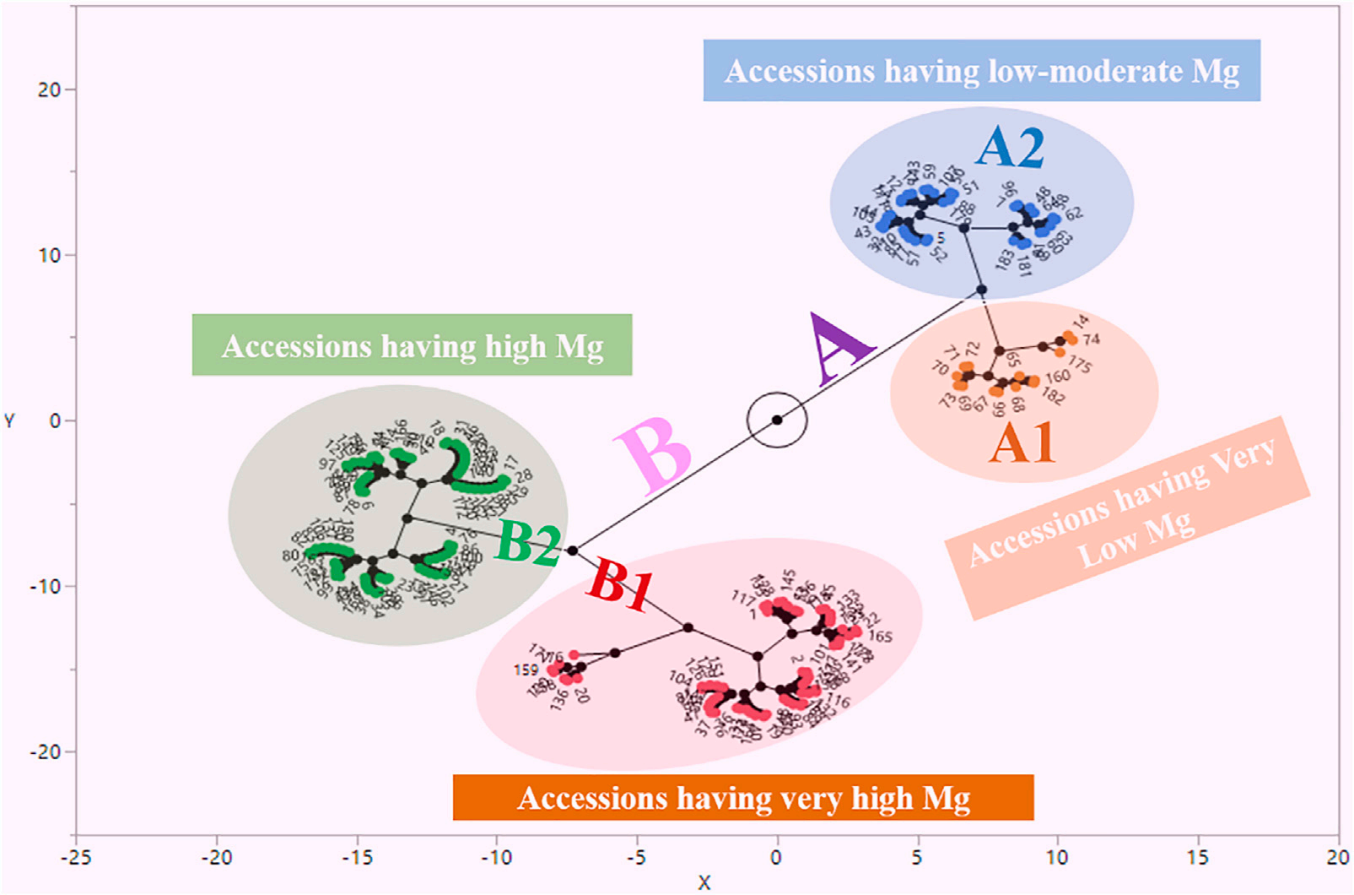
Constellation plot for magnesium content in Turkish common bean germplasm.

Mg contents variations were also observed at the geographical provinces from where the studied germplasm was collected. By taking the mean of two locations, we observed that maximum Mg contents were reflected by landraces from Bolu (1.13 mg kg^−1^), while landraces from Bitlis province were found poor (0.76 mg kg^−1^) in seed Mg contents (Figure 2). We performed the stability analysis using the mean of two environments and succeeded in identifying eight landraces with high Mg contents in common bean seeds (Table 3). The constellation plot separated the studied germplasm into two main populations A and B (Figure 3). Population A clustered accessions with poor Mg contents compared to population B. Population A was further subdivided into two subpopulations A1 and A2. Population B was further subdivided into B1 and B2, while subpopulation B2 clustered accessions rich in Mg contents.

**TABLE 3:** The most stable common bean accessions for Mg contents.

Landraces	Mean Mg contents	s^2^d_i_	b_i_	CVi
Hakkari-28	0.94	0	0	0
Bitlis-35	1.05	0	0	0
Bitlis-69	1.17	0	0	0
Bursa-1	1.13	2.31E-06	0.199184	0.509427
Duzce-9	1.13	2.45E-06	0.197323	0.512441
Hakkari-69	1.05	2.31E-06	0.199184	0.548117
Van-33	1.05	2.31E-06	0.199184	0.548117
Hakkari-65	1.04	2.45E-06	−0.19732	0.553371

CVi, Coefficient of variance, s^2^d_i_ = Deviation from regression, b_i_ = Regression coefficient 691.

### 3.2 Genomic Regions and Putative Genes Associated With Mg Contents

**FIGURE 4:**
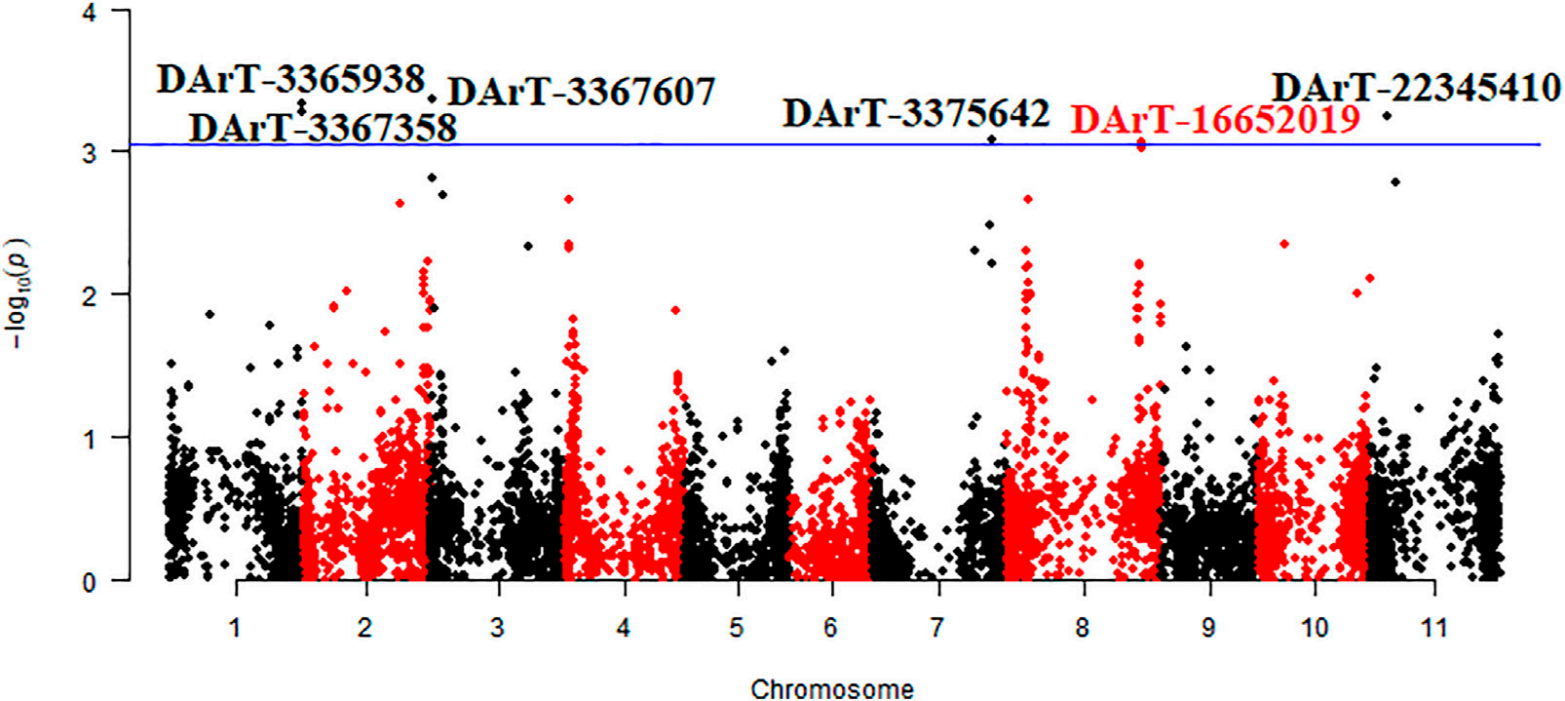
Manhattan plot of marker trait association for magnesium content in Turkish common bean germplasm.

**FIGURE 5:**
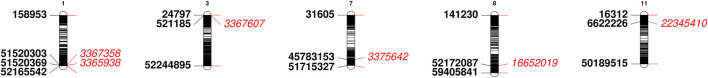
Physical map of identified DArTseq markers having association with seed Mg contents in Turkish common bean germplasm.

The mean data of two environments (Bolu and Sivas) were used for the identification of chromosomal regions associated with seed Mg contents and a total of 6 DArTseq markers were found statistically significant for Mg contents in common bean seeds (Table 4 and Figure 4). A total of two markers (DArT-3365938 and DArT-3367358) were present on chromosome Pv01, while rest of four markers (one marker for each chromosome) were present on chromosome Pv03, Pv07, Pv08 and Pv11. Among these identified markers, DArT-3367607 marker contributed in maximum (7.5%) variations. A total of five putative candidate genes were also identified from sequences reflecting homology to six identified DArTseq markers. Vigun01g245600 putative gene was predicted as a putative gene for DArT-3365938 and DArT-3367358 markers. The constructed physical map of identified markers revealed a very narrow region for 0.00000101 Mbp for DArT-3367358 and DArT-3365938 markers (Figure 5).

**TABLE 4:** Chromosomal regions associated with seed Mg contents in Turkish common bean germplasm.

Marker	Chromosome	Position (bp)	*p*-value	R2%	Putative gene (bp)
DArT-3367607	3	521,185	4.29E-04	7.5	Phvul.003G001300
DArT-3365938	1	51,520,369	4.62E-04	7.4	Vigun01g245600
DArT-3367358	1	51,520,303	5.31E-04	7.3	Vigun01g245600
DArT-22345,410	11	6,622,226	5.61E-04	6.9	Phvul.011G071900
DArT-3375642	7	45,783,153	8.19E-04	6.5	Phvul.005G079500
DArT-16652019	8	52172087	8.65E-04	6.7	Phvul.008G185200

## 4 Discussion

Despite the well-known role of Mg for human health and plant metabolism, little or negligible research has been conducted on this important element. As discussed earlier, Mg is considered a “Forgotten element” and there is an utmost need to characterize the germplasm of different crops in order to identify the accessions with high Mg contents in the edible parts of crops to eradicate the malnutrition of this important mineral element in the human population. Common bean is one of the most important legume crops used frequently in the diets of the human populations of the developing and least developed countries, and therefore crops like common bean are considered as “poor man’s meat”. Moreover, common bean is referred to as a “grain of hope” as its seeds are rich sources of various mineral elements crucial for human health (Nadeem et al., 2021a). The present work was done with an objective of characterizing the mini core collection of common bean germplasms from Turkey, an important area of diversity for common bean, to find the promising accessions with high Mg contents in the common bean seeds and also to unlock the chromosomal region associated with this mineral element and to discuss whether the genomic regions identified here are novel QTLs or whether they fall into the same genetic region reported earlier.

Analysis of variance (ANOVA) revealed significant differences (*p* < 0.05) for Mg contents in both environments, however the genotype x environment was non-significant (Table 1). Heritability analysis showed a moderate level of heritability. These results were found in line with the very recent study of Delfini et al. (2021). They also observed significant effects within the environment, while there was no Genotype x environment (G x E) interactions. Moreover they also found a low range of Heritability (0.18–0.47). During this study, plentiful variation (0.330–1.520 mg kg^−1^) was seen for seed Mg contents (Table 2). Moreover, frequency distribution clearly depicted that most of the accessions have high (above than 0.9 mg kg-1) Mg contents (Figure 1). The mean and range of Mg contents in the studied germplasm (Table 2) were found higher than in our previous study (Yeken et al., 2019). This could be because the germplasm used in the present study was different from our previous studies and the size of the germplasm in the earlier study was small compared with the germplasm used in the current study. The amount of the Mg contents in common bean accessions used in this study was comparable or slightly lower than the previous studies. Very recently, Gunjača et al. (2021) aimed to explore marker-trait association in common bean germplasm for mineral contents and reported Mg contents in a range of 0.13–0.24% in dry weight (DW). Palčić et al. (2018) also reported Mg content in a range of 0.17–0.2% DW, with the average of 0.18%. Delfini et al., 2021 analyzed 178 Mesoamerican accessions in three different conditions and found the Mg content to range between 164—290 mg/100 g. Augustin et al. (1981) used samples from nine classes of common bean germplasm from United States of America, and found that raw common beans contain 16–230 mg magnesium whereas cooked common bean have slightly reduced Mg content of 130–220 mg per 100 g dry weight. Ray et al. (2014) used 10 common bean cultivars grown in Saskatchewan, Canada in six locations and found the Mg content in a range of 184.5–238.3 mg/100 g. In another study, seven common bean genotypes from Manitoba and Saskatchewan, Canada were evaluated and Mg contents varied from 143.0–199.5 mg/100 g (Wang et al., 2010). In their assay Akond et al. (2011) used 29 common bean genotypes from CIAT (International Center for Tropical Agriculture), United States, India and Brazil and found the Mg content 0.647–1.105 mg/100 g. Brigide et al. (2014), in their study, used four biofortified and one control common bean variety. The Mg content in 100 g seeds was measured 11.2–17.3 mg in the raw treatment and 16.1–17.1 mg in the macerated/cooked treatment. Existence of good variation in the Turkish common bean germplasm for Mg contents could be successfully used for breeding the common bean with a higher Mg composition of seeds.

The world is facing the disaster of climate change, and therefore selection of the stable genotypes is one of the most important criteria for effective breeding programs. The environment has always had a magnificent effect on the genotypes, therefore genotypes with maximum stability are identified with the least environmental effect. In this study, eight common bean accessions reflecting the highest stability for seed Mg contents were evaluated and can be used for future common bean breeding programs (Table 3). These stable accessions were evaluated according to coefficient of variance, deviation from regression, and regression coefficient as described in our previous study (Nadeem et al., 2021b). According to Francis and Kannenberg (1978), accessions with a low coefficient of variance have minimal environmental variance and can be used as the most stable accessions. All of the identified and stable accessions reflected a good range of mean Mg contents (0.94–1.17 mg kg^−1^). Therefore, these identified accessions can serve as a source of genetic resource for the biofortification of common bean regarding Mg contents.

The germplasm used in this research was collected from various geographical provinces with the different topography, climate, and agricultural practices of Turkey. Therefore, we also analyzed the germplasm according to their geographical provinces (Figure 2). Accessions from the Bolu province showed the highest Mg contents while the Bitlis province reflected the lowest Mg contents. Bolu is located in the west Black sea region of the Turkey, while the Bitlis province is located in the East Anatolian region of Turkey. During our previous study regarding morpho-agronomic characterization of Turkish common bean germplasm, we found that accessions from the Bolu province have higher 100 seeds weight compared to accessions from Bitlis. It is clearly understandable by making a comparison of seed Mg contents with 100 seeds weight at the province level from our previous study (Nadeem et al., 2020a), most of the provinces with higher 100 seeds weight reflected higher Mg contents. Keeping in view these findings, we can postulate that accessions with higher seed weight may have higher Mg contents (Nadeem and Baloch’s personal perception).

To see the pattern of variation, clustering analysis was performed to observe the grouping of studied germplasm. The studied germplasm was divided into two populations i.e., A and B on the basis of their Mg contents (Figure 3). Most of the accessions with comparatively low Mg contents were clustered in group A. Population A was further subdivided into A1 and A2, while the A1 subpopulation clustered those accessions with very low Mg contents. Mean minimum Mg contents (0.330 mg kg^−1^) were observed for Nigde-Dermasyon landraces that were also present in sub-population A1. Sub-population A2 clustered accessions had low to moderate Mg contents (0.60–0.90 mg kg^−1^). Population B clustered accessions had high Mg contents. Sub-population B1 clustered accessions had very high Mg contents compared to B2. Most of the accession present in B1 sub-population reflected Mg contents above 1 mg kg^−1^. Nigde-Derinkuyu landrace reflected maximum mean Mg contents during this study and it was present in B1 group. Accessions present in the B2 group reflected higher Mg contents compared to population A and lower than sub-population B1. As accessions present in B1 sub-population were rich in Mg contents. It is recommended to utilize the accessions of this sub-population for the breeding perspective of common bean.

### 4.1 Marker-Trait Association

A total of six DArTseq markers showed significant association with seed Mg contents in Turkish common bean germplasm (Table 4 and Figure 4). DArT-3367607 was the only marker present at Pv03 and contributed in highest phenotypic variations (7.5%). A total of two DArTseq markers (DArT- 3365938 and DArT-3367358) showed their distribution on chromosome Pv01 and reflected 7.4 and 7.3% variations respectively. Similarly, one statistically significant marker on each of chromosome 333 Pv07 (DArT-3375642), Pv08 (DArT-16652019) and Pv011 (DArT-22345,410) was observed with phenotypic variation of 6.5, 6.7 and 6.9 respectively (Table 4). Very recently, Delfini et al. (2021) performed GWAS analysis to identify quantitative trait nucleotides (QTNs) for mineral contents in common bean diversity panel from Brazil. They reported distribution of QTNs for Mg contents on various chromosomes of common bean. They reported S03_552367 as a QTN on chromosome Pv03 at the position of 552,367 bp. Similarly, DArT-3367607 marker identified in this study was present on the chromosome Pv03 at the position of 521185 bp. Both markers were present within a very narrow region of 0.031182 Mbp. Similarly, Gunjaca et al. (2021) identified only one marker on chromosome Pv08 for Mg contents in Croatian common bean germplasm and phenotypic variation explained by this marker was low when compared with this study. Their marker chromosomal position was 50,916,423 bp, while our identified marker on the same chromosome was at 52172087 bp. Both markers were present in a region of 1.255 Mbp. Blair et al. (2016) also reported the distribution of QTLs for Mg contents on Pv07, Pv08, and Pv10. They stated that the identified QTL (P9DB1D) present on Pv07 chromosome was in the region of the *Phs* locus which has been found to be a very important region with multiple genes that influenced Fe and Zn concentration (Blair et al., 2009). As Blair et al. (2016) also found QTL present on Pv08, they stated that this QTL near the marker Bng96 aligned with a previous QTL for Fe contents (Blair et al., 2012). Casañas et al. (2013) reported a QTL (Mg7^xc^) for Mg contents in common bean on Pv07 and reported *P* gene as a closest marker to this QTL. They also reported that this *P* gene has association with calcium, ashes, dietary fiber, and uronic acid contents in common bean. Some markers identified in this study were found in the same chromosomal regions reported by Delfini et al. (2021) and Gunjaca et al. (2021). Therefore, it could be further studied for validation through candidate gene association mapping. Most of the markers found in this study could be associated with novel/new QTLs that could be present in Turkish germplasm and can be used for marker-assisted breeding of common bean. Additionally, the physical map disclosed that two markers i.e. DArT-3367358 and DArT-3365938 with association for seed Mg contents in common bean were present on the chromosomes Pv01 at 51.52 Mbp and 52.16 Mbp respectively (Figure 5). Both markers were present in a very narrow chromosomal region with a distance of 0.00000101 Mbp. Therefore, this region containing both markers with association for seed Mg contents should be considered for future common bean breeding.

During the present investigation, sequences of the identified markers were used to BLAST-search against the common bean genome in the legume information system (LIS: https://legumeinfo.org/) and putative genes were investigated. Phvul.003G001300 was identified as a putative gene for DArT-3367607 marker. This gene encodes for Pentatricopeptide repeat (PPR) superfamily protein. This family is characterized by tandem 30–40 amino acid sequence motifs and considered one of the largest protein families in land plants (Xing et al., 2018). Zhang et al. (2020) stated that this family is involved in the post-transcriptional processing of RNA in chloroplasts and mitochondria, which is very important for plant development and evolutionary adaption. Previous studies confirmed that mitochondria have the capability of accumulation of Mg and ultimately act as an important intracellular Mgstore (Kubota et al., 2005; Shindo et al., 2011). Vigun01g245600 present on chromosome 01 of *Vigna unguiculata* was found to be a putative gene for DArT-3365938 and DArT-3367358. This gene encodes for Ankyrin repeat family protein, which is considered to be one of the largest protein families. This protein family is involved in various processes like plant growth and development, hormone response, and contributes significantly to resistance to abiotic and biotic stresses (Lopez-Ortiz et al., 2020). Zhao et al. (2020) revealed the role of this protein family in salt and drought tolerance in Arabidopsis and Soybean. Verbruggen and Hermans (2013) clearly explored the role of Mg in various physiological and biochemical processes in plants. Phvul.011G071900 resulted as a putative gene for DArT-22345410 that encodes for DOF zinc finger protein. DOF is a family of transcription factors that contributes significantly to various fundamental processes like seed germination, seed maturation phytohormone production and response to light (Kang et al., 2016). Zhang et al. (2020) clearly explored the role of Mg for yield and seed germination traits in wax gourd crop. They concluded that seeds derived from Mg-sufficient plants were more vigorous and have earlier emergence, better seedling establishment, and better development compared to the seeds collected from Mg deficient plants. Phvul.005G079500 was found to be a putative gene for DArT-3375642 and this gene encodes for zinc finger (Ran-binding) family protein. Zinc figure proteins comprise one of the largest transcription factor families and play a significant role in various abiotic stress resistances (Han et al., 2020). Phvul.008G185200 was found as a putative gene for DArT-16652019 marker and this gene encodes for mate efflux family protein (MATE). Members of this family are present abundantly in plants and contribute to growth and developmental processes (Chen et al., 2015). Transporters of this family are directly or indirectly involved in detoxification of toxic compounds, heavy metals resistance, disease resistance, and response to hormone regulation (Wu et al., 2014; Santos et al., 2017). Previous studies explored the role of this protein family against aluminum toxicity (Liu et al., 2016; Wang et al., 2017). Bose et al. (2011) comprehensively explored the role of Mg in reducing aluminum toxicity in plants.

## 5 Conclusion

The present investigation provided a deep insight into the existence of the wide range of Mg contents diversity in Turkish common bean germplasm. genotype × environment interaction showed non-significant effects, while a moderate level of heritability was observed for the studied trait. Accessions from Nigde province showed maximum range of variation in seed Mg contents. Some stable bean accessions were also identified which can be explored in the crossing program as parents for developing bean varieties with stable Mg contents under various environmental conditions. The present investigation reported six DArTseq markers with association for seed Mg contents. Identified markers with association for Mg contents were found within a narrow region in which previous markers for Mg contents have been reported by earlier studies. Keeping this in view, identified markers in this study should be validated along with previously reported markers. After validating these markers, they can be effectively used in marker assisted selection for breeding bean with higher Mg contents. We are confident that the information presented in this study will be helpful for common bean breeding regarding Mg contents.

## Data Availability

The original contributions presented in the study are included in the article/Supplementary Material, further inquiries can be directed to the corresponding authors.
