# Erratum: Magnesium-a Forgotten Element: Phenotypic Variation and Genome Wide Association Study in Turkish Common Bean Germplasm

**DOI:** 10.3389/fgene.2022.964612

**Published:** 2022-07-15

**Authors:** Frontiers Production Office

**Affiliations:** Frontiers Media SA, Lausanne, Switzerland

**Keywords:** *Phaseolus vulgaris*, food legume, Mg contents, DArTseq, GWAS

Due to a production error, an author name was incorrectly spelled as “Gönül Cömertpa”. The correct spelling is “Gönül Cömertpay”.

The publisher apologizes for this mistake. The original version of this article has been updated.

