# Supplementary material for: Magnesium- a Forgotten Element: Phenotypic Variation and Genome Wide Association Study in Turkish Common Bean Germplasm
Source: Front Genet. 2022 May 2;13:848663. doi: 10.3389/fgene.2022.848663 (PMC9108430; doi:10.3389/fgene.2022.848663)
Supplement: Supplementary file 2 [file Table2.DOCX]

**Supplementary Table 2:** Diversity of Mg contents in the seeds of Turkish common bean germplasm

| Genotypes | Bolu2018 | Sivas2018 | Mean |
| --- | --- | --- | --- |
| Bingol-1 | 1.13 | 1.27 | 1.20 |
| Bingol-6 | 0.99 | 1.16 | 1.07 |
| Bingol-7 | 1.00 | 1.18 | 1.09 |
| Bingol-11 | 0.93 | 1.06 | 0.99 |
| Bingol-16 | 0.69 | 0.86 | 0.77 |
| Bingol-18 | 0.84 | 0.91 | 0.88 |
| Bingol-25 | 0.65 | 0.75 | 0.70 |
| Bingol-33 | 0.80 | 0.82 | 0.81 |
| Bingol-36 | 1.08 | 1.13 | 1.11 |
| Bingol-44 | 0.85 | 0.94 | 0.90 |
| Bingol-45 | 0.97 | 1.04 | 1.00 |
| Bingol-52 | 0.77 | 0.83 | 0.80 |
| Bingol-53 | 0.74 | 0.81 | 0.78 |
| Bingol-58 | 0.37 | 0.44 | 0.41 |
| Bingol-60 | 1.00 | 1.07 | 1.03 |
| Bingol-61 | 1.04 | 1.06 | 1.05 |
| Bingol-63 | 0.87 | 0.96 | 0.91 |
| Bingol-65 | 0.86 | 0.95 | 0.90 |
| Hakkari-7 | 0.99 | 1.07 | 1.03 |
| Hakkari-11 | 1.22 | 1.27 | 1.24 |
| Hakkari-12 | 1.06 | 1.18 | 1.12 |
| Hakkari-13 | 1.12 | 1.14 | 1.13 |
| Hakkari-16 | 0.89 | 0.97 | 0.93 |
| Hakkari-20 | 0.83 | 0.85 | 0.84 |
| Hakkari-23 | 0.84 | 0.90 | 0.87 |
| Hakkari-28 | 0.94 | 0.94 | 0.94 |
| Hakkari-31 | 0.97 | 1.03 | 1.00 |
| Hakkari-37 | 0.88 | 0.93 | 0.91 |
| Hakkari-38 | 0.86 | 0.95 | 0.91 |
| Hakkari-39 | 0.79 | 0.86 | 0.83 |
| Hakkari-43 | 0.86 | 0.95 | 0.90 |
| Hakkari-44 | 0.72 | 0.77 | 0.75 |
| Hakkari-51 | 1.01 | 1.06 | 1.04 |
| Hakkari-55 | 0.93 | 0.95 | 0.94 |
| Hakkari-63 | 0.87 | 0.96 | 0.91 |
| Hakkari-65 | 1.05 | 1.04 | 1.04 |
| Hakkari-69 | 1.05 | 1.06 | 1.05 |
| Hakkari-71 | 0.95 | 0.94 | 0.94 |
| Hakkari-76 | 0.89 | 0.96 | 0.93 |
| Tokat-83 | 0.98 | 1.08 | 1.03 |
| Maras-92 | 0.82 | 0.84 | 0.83 |
| Bitlis-5 | 0.95 | 0.98 | 0.97 |
| Bitlis-14 | 0.74 | 0.76 | 0.75 |
| Bitlis-16 | 0.75 | 0.77 | 0.76 |
| Bitlis-22 | 1.06 | 1.17 | 1.11 |
| Bitlis-25 | 0.86 | 0.94 | 0.90 |
| Bitlis-35 | 1.05 | 1.05 | 1.05 |
| Bitlis-40 | 0.73 | 0.66 | 0.69 |
| Bitlis-46 | 0.66 | 0.64 | 0.65 |
| Bitlis-48 | 0.80 | 0.77 | 0.79 |
| Bitlis-53 | 0.81 | 0.78 | 0.79 |
| Bitlis-66 | 0.79 | 0.76 | 0.77 |
| Bitlis-69 | 1.17 | 1.17 | 1.17 |
| Bitlis-76 | 0.84 | 0.78 | 0.81 |
| Bitlis-79 | 0.92 | 0.87 | 0.90 |
| Bitlis-81 | 0.85 | 0.86 | 0.86 |
| Bitlis-90 | 0.79 | 0.74 | 0.77 |
| Bitlis-94 | 0.69 | 0.67 | 0.68 |
| Bitlis-97 | 0.82 | 0.74 | 0.78 |
| Bitlis-103 | 0.69 | 0.65 | 0.67 |
| Bitlis-105 | 0.69 | 0.64 | 0.67 |
| Bitlis-111 | 0.68 | 0.67 | 0.68 |
| Bitlis-114 | 0.69 | 0.67 | 0.68 |
| Bitlis-115 | 0.72 | 0.70 | 0.71 |
| Bitlis-117 | 0.64 | 0.59 | 0.61 |
| Bitlis-118 | 0.57 | 0.57 | 0.57 |
| Bitlis-119 | 0.58 | 0.55 | 0.57 |
| Bitlis-120 | 0.59 | 0.59 | 0.59 |
| Bitlis-121 | 0.55 | 0.51 | 0.53 |
| Bitlis-124 | 0.55 | 0.54 | 0.54 |
| Malatya-3 | 0.56 | 0.55 | 0.55 |
| Malatya-13 | 0.57 | 0.53 | 0.55 |
| Malatya-14 | 0.54 | 0.51 | 0.53 |
| Malatya-18 | 0.47 | 0.46 | 0.47 |
| Malatya-25 | 0.96 | 0.97 | 0.96 |
| Malatya-28 | 1.04 | 0.95 | 0.99 |
| Malatya-32 | 0.83 | 0.72 | 0.77 |
| Malatya-33 | 0.91 | 0.85 | 0.88 |
| Malatya-45 | 0.94 | 0.85 | 0.90 |
| Malatya-50 | 0.97 | 0.95 | 0.96 |
| Malatya-51 | 1.10 | 1.03 | 1.06 |
| Malatya-52 | 0.97 | 0.96 | 0.96 |
| Malatya-59 | 0.97 | 0.94 | 0.96 |
| Malatya-71 | 1.13 | 1.06 | 1.09 |
| Tunceli-1 | 0.81 | 0.74 | 0.77 |
| Tunceli-5 | 1.01 | 0.97 | 0.99 |
| Tunceli-11 | 0.90 | 0.86 | 0.88 |
| Van-1 | 0.82 | 0.75 | 0.79 |
| Van-11 | 0.89 | 0.87 | 0.88 |
| Van-13 | 0.86 | 0.84 | 0.85 |
| Van-17 | 0.99 | 0.97 | 0.98 |
| Van-19 | 0.94 | 0.86 | 0.90 |
| Van-25 | 1.11 | 1.08 | 1.09 |
| Van-27 | 0.89 | 0.83 | 0.86 |
| Van-33 | 1.05 | 1.06 | 1.05 |
| Van-36 | 0.74 | 0.66 | 0.70 |
| Van-42 | 0.89 | 0.86 | 0.87 |
| Van-47 | 0.94 | 0.87 | 0.91 |
| Van-51 | 1.00 | 0.93 | 0.97 |
| Van-59 | 1.03 | 0.95 | 0.99 |
| Van-64 | 1.09 | 1.05 | 1.07 |
| Van-65 | 1.04 | 0.96 | 1.00 |
| Van-68 | 0.95 | 0.90 | 0.93 |
| Elazig-2 | 1.05 | 1.07 | 1.06 |
| Elazig-7 | 0.77 | 0.73 | 0.75 |
| Elazig-9 | 0.96 | 0.97 | 0.96 |
| Elazig-10 | 0.80 | 0.76 | 0.78 |
| Elazig-14 | 1.12 | 1.04 | 1.08 |
| Elazig16 | 0.93 | 0.85 | 0.89 |
| Elazig-25 | 0.82 | 0.72 | 0.77 |
| Elazig-27 | 0.76 | 0.75 | 0.76 |
| Elazig-29 | 0.99 | 0.94 | 0.97 |
| Elazig-30 | 0.83 | 0.97 | 0.90 |
| Elazig-34 | 0.75 | 0.96 | 0.86 |
| Elazig-36 | 0.99 | 1.16 | 1.07 |
| Elazig-39 | 0.99 | 1.17 | 1.08 |
| Mus-1 | 1.12 | 1.27 | 1.19 |
| Mus-2 | 1.09 | 1.24 | 1.16 |
| Mus-7 | 0.93 | 1.04 | 0.99 |
| Mus-10 | 1.20 | 1.36 | 1.28 |
| Mus-15 | 0.85 | 0.97 | 0.91 |
| Mus-18 | 0.99 | 1.15 | 1.07 |
| Mus-22 | 1.06 | 1.24 | 1.15 |
| Mus-27 | 0.85 | 0.96 | 0.90 |
| Mus-28 | 0.79 | 0.96 | 0.87 |
| Mus-34 | 0.96 | 1.17 | 1.06 |
| Mus-39 | 0.91 | 1.06 | 0.99 |
| Mus-41 | 1.14 | 1.22 | 1.18 |
| Mus-42 | 0.99 | 1.14 | 1.06 |
| Mus-43 | 1.00 | 1.18 | 1.09 |
| Mus-46 | 0.83 | 0.97 | 0.90 |
| Mus-48 | 1.00 | 1.15 | 1.08 |
| Mus-49 | 1.05 | 1.17 | 1.11 |
| Mus-50 | 0.86 | 1.04 | 0.95 |
| Mus-51 | 1.12 | 1.26 | 1.19 |
| Mus-52 | 1.16 | 1.34 | 1.25 |
| Mus-53 | 0.88 | 1.05 | 0.96 |
| Sivas-3 | 0.91 | 1.07 | 0.99 |
| Sivas-4 | 0.98 | 1.16 | 1.07 |
| Sivas-7 | 0.84 | 0.95 | 0.90 |
| Sivas-12 | 1.06 | 1.23 | 1.14 |
| Sivas-13 | 0.85 | 0.96 | 0.91 |
| Sivas-16 | 0.74 | 0.86 | 0.80 |
| Sivas-17 | 0.95 | 1.15 | 1.05 |
| Sivas-18 | 1.09 | 1.27 | 1.18 |
| Sivas-44 | 0.94 | 1.05 | 1.00 |
| Sivas-62 | 0.96 | 1.15 | 1.05 |
| Sivas-68 | 1.04 | 1.15 | 1.09 |
| Sivas-69 | 0.79 | 0.94 | 0.87 |
| Sivas-70 | 0.86 | 1.07 | 0.96 |
| Bilecik-1 | 0.96 | 1.15 | 1.06 |
| Bilecik-2 | 0.88 | 1.06 | 0.97 |
| Bilecik-6 | 1.03 | 1.16 | 1.10 |
| Bilecik-7 | 0.79 | 0.94 | 0.87 |
| Bilecik-10 | 1.04 | 1.18 | 1.11 |
| Balikesir-3 | 1.07 | 1.29 | 1.18 |
| Balikesir-4 | 0.80 | 0.95 | 0.88 |
| Balikesir-5 | 1.16 | 1.34 | 1.25 |
| Balikesir-6 | 1.18 | 1.38 | 1.28 |
| Balikesir-17 | 0.55 | 0.66 | 0.60 |
| Balikesir-18 | 0.93 | 1.07 | 1.00 |
| Balikesir-19 | 0.85 | 0.96 | 0.91 |
| Bal?ikesir-20 | 0.92 | 1.05 | 0.98 |
| Duzce-1 | 0.99 | 1.07 | 1.03 |
| Duzce-9 | 1.12 | 1.13 | 1.13 |
| Yalova-13 | 0.83 | 0.85 | 0.84 |
| Yalova-20 | 0.97 | 1.04 | 1.01 |
| Yalova-21 | 1.05 | 1.10 | 1.08 |
| Erzincan-1 | 0.92 | 0.96 | 0.94 |
| Erzincan-3 | 0.89 | 0.94 | 0.91 |
| Erzincan-4 | 1.25 | 1.37 | 1.31 |
| Erzincan-5 | 0.87 | 0.96 | 0.91 |
| Bursa-1 | 1.13 | 1.14 | 1.13 |
| Bursa-22 | 1.00 | 1.06 | 1.03 |
| Nigde-Dermasyon | 0.32 | 0.34 | 0.33 |
| Nigde-Derinkuyu | 1.49 | 1.55 | 1.52 |
| Civiril-Bolu | 1.12 | 1.16 | 1.14 |
| Akman | 0.91 | 0.97 | 0.94 |
| Goynuk | 0.77 | 0.81 | 0.79 |
| Karacesehir | 0.94 | 0.99 | 0.96 |
| Onceler | 0.66 | 0.65 | 0.64 |
| Goksun | 0.61 | 0.62 | 0.60 |
| Akdag | 0.64 | 0.65 | 0.63 |

× Commercial cultivars, - Unknown
